# Cardioversion and Risk of Adverse Events with Dabigatran versus Warfarin—A Nationwide Cohort Study

**DOI:** 10.1371/journal.pone.0141377

**Published:** 2015-10-29

**Authors:** Jannik Langtved Pallisgaard, Tommi Bo Lindhardt, Morten Lock Hansen, Anne-Marie Schjerning, Jonas Bjerring Olesen, Laila Staerk, Christian Torp-Pedersen, Gunnar Hilmar Gislason

**Affiliations:** 1 Department of Cardiology, Copenhagen University Hospital Gentofte, Hellerup, Denmark; 2 Faculty of Health and Medical Sciences, University of Copenhagen, Copenhagen, Denmark; 3 The Heart Centre, Copenhagen University Hospital Rigshospitalet, Copenhagen, Denmark; 4 Institute of Health, Science and Technology, Aalborg University, Aalborg, Denmark; 5 The National Institute of Public Health, University of Southern Denmark, Copenhagen, Denmark; 6 The Danish Heart Foundation, Copenhagen, Denmark; Providence VA Medical Center and Brown University, UNITED STATES

## Abstract

**Aim:**

Cardioversion can rapidly and effectively restore sinus rhythm in patients with persistent atrial fibrillation. Since 2011 dabigatran has been available as an alternative to warfarin to prevent thromboembolic events in patients with non-valvular atrial fibrillation undergoing cardioversion. We studied time to cardioversion, risk of adverse events, and risk of readmission with atrial fibrillation after cardioversion according to anticoagulation therapy.

**Methods and Results:**

Through the nationwide Danish registries we included 1,230 oral anticoagulation naïve patients with first time non-valvular atrial fibrillation and first time cardioversion from 2011 to 2012; 37% in the dabigatran group (n = 456), and 63% in the warfarin group (n = 774). Median time to cardioversion was 4.0 (interquartile range [IQR] 2.9 to 6.5) and 6.9 (IQR 3.9 to 12.1) weeks in the dabigatran and warfarin groups respectively, and the adjusted odds ratio of cardioversion within the first 4 weeks was 2.3 (95% confidence interval [CI] 1.7 to 3.1) in favor of dabigatran. The cumulative incidence of composite endpoint of stroke, bleeding or death were 2.0% and 1.0% at 30 weeks in the warfarin and dabigatran groups respectively, with an adjusted hazard ratio of 1.33 (95% CI 0.33 to 5.42). Cumulative incidence of readmission with atrial fibrillation after 30 weeks were 9% and 11% in the warfarin and dabigatran groups, respectively, and an adjusted hazard ratio of 0.66 (95% CI 0.41 to 1.08).

**Conclusion:**

Anticoagulation treatment with dabigatran allows shorter time to cardioversion for atrial fibrillation than warfarin, and appears to be an effective and safe alternative treatment strategy to warfarin.

## Introduction

Atrial fibrillation is the most frequent cardiac arrhythmia with a prevalence of about 1–2% in the general population.[1] Cardioversion can be used to restore sinus rhythm in patients with persistent atrial fibrillation, but requires oral anticoagulation for at least 3 weeks in patients with atrial fibrillation duration above 48 hours. In non-anticoagulated patients the peri-procedural risk of a stroke event associated with cardioversion is between 5 and 7%, but treatment with warfarin reduces incidence of thromboembolic events to between 0.5 and 1.6%,[2–5] hence international guidelines recommend at least 3 consecutive weeks of effective anticoagulation before cardioversion, followed by at least 4 weeks of anticoagulation.[1,6] Since August 22, 2011 dabigatran has been an alternative to warfarin in Denmark as oral anticoagulation therapy in patients with non-valvular atrial fibrillation requiring cardioversion.[7,8] The main objective of this study was to investigate the time to cardioversion in anticoagulation naïve patients with first-time atrial fibrillation, according to initiated anticoagulation therapy with either dabigatran or warfarin. Further, we assessed the risk of cardiovascular adverse events or death and the risk of readmission with atrial fibrillation after cardioversion according to anticoagulation treatment strategy.

## Methods

In Denmark all residents are at birth or immigration provided with a permanent and unique civil registration number that enables individual level linkage between administrative registries.

The Danish National Patient Register holds information on all discharges from hospitals in Denmark since 1978.[9] Each hospitalization is at discharge coded with one primary and, if appropriate, one or more secondary diagnoses according to the International Classification of Diseases, the 8th revision (ICD-8) until 1994 and the 10th revision (ICD-10) thereafter.

Data on pharmacy prescriptions claims were identified from the Danish Registry of Medicinal Product Statistics that keeps records on all drug prescriptions dispensed from Danish pharmacies since 1995. Each drug dispensing is registered according to an international classification of drugs, the Anatomical Therapeutic Chemical (ATC) system, as well as the date of dispensing, quantity dispensed, strength, formulation, and affiliation of the physician issuing the prescription. The partial reimbursement of drug expenses by the Danish health care system requires all pharmacies to register each drug dispensing in the National Prescription Registry.

Causes of death were obtained from the National Causes of Death Register, in which both underlying and immediate causes of death are recorded using the International Classification of Diseases (ICD).[10],[9]

### Study population and follow up

Inclusion criteria were: First time discharge coding diagnosis with non-valvular atrial fibrillation between August 22, 2011 and December 31, 2012, no prior anticoagulation treatment and cardioversion performed after anticoagulation treatment initiation. Both in and outpatients were eligible to enter the study. Valid data on onset date of atrial fibrillation and type of atrial fibrillation (paroxysmal, persistent or permanent) was not available in this study, because of this first day of anticoagulation treatment was chosen as day of entry in the study. Patients who received warfarin entered the warfarin group, and patients receiving dabigatran were assigned to the dabigatran group regardless of treatment dosage. We excluded patients with prior anticoagulation treatment or treated with other anticoagulation than dabigatran or warfarin.

Patients were followed until end of study (December 31. 2012), change of anticoagulation treatment regime, time of death, or date of an endpoint of interest.

### Study cohort

Newly onset atrial fibrillation after August 22, 2011 was identified using ICD-10 codes for atrial fibrillation. Non-valvular atrial fibrillation was identified excluding patients with a diagnosis of rheumatic valvular disease by using ICD-8 and ICD-10 codes, or a history with prosthetic heart valve replacement, using the specific Nordic procedure codes for valve replacements. Study start was the date of a claimed prescription of dabigatran or warfarin using ATC codes. Information on age and gender came from the Danish Civil Registration System. Comorbidities were identified using ICD-8 and ICD-10 codes for: heart failure, chronic kidney disease, peripheral arterial disease, stroke, ischemic heart disease, chronic pulmonary obstructive disease, bleeding, liver disease and cancer. Glucose lowering medication and antihypertensive drugs were identified by ATC codes and used as proxies for diabetes mellitus and hypertension, respectively The method used to identify hypertension in this study has a positive predictive value of 80.0%, and a specificity of 94.7%.[11] ATC codes were also used to identify: concomitant drug: verapamil, amiodarone, dronedarone, flecainide, digoxin, non-steroidal anti-inflammatory drugs (NSAID), antiplatelet and acetylsalicylic acid (Appendix 1). Cardioversion and trans esophageal echocardiogram was identified by Danish national non-surgical procedure codes. Analyzing for non-inferiority between warfarin and dabigatran treatment between warfarin and dabigatran treatment, would have required approximately 30.000 patients which was not possible with current study design.

### Study outcomes

The study outcome was time between date of claimed prescription of dabigatran or warfarin to date of cardioversion and proportion of patients undergoing cardioversion within the first 4 weeks All procedure of cardioversions are registered holding information on the time and date the cardioversion was performed (Appendix 1). The risk of readmission with atrial fibrillation within thirty weeks from cardioversion and the risk of a composite endpoint of stroke, major bleeding and death within thirty weeks after cardioversion were analyzed. Stroke and bleeding were identified using ICD-10 codes, and information on death came from National Causes of Death Register. (Appendix 1)

### Statistical analysis

Baseline characteristics were presented as medians with interquartile range (IQR) or frequencies and percentages. Differences between baseline characteristics were compared by Chi-square, Fishers, or Kruskal-Wallis test as appropriate. Time from claimed prescription of anticoagulation treatment to date of cardioversion was graphically as medians with IQR in a jitter plot and tested for significance by Welch Two Sample t-test. Chance of cardioversion within the first 4 weeks was analyzed with multivariable logistic regression analysis adjusted for age, sex, comorbidities and concomitant drugs. Predicted probabilities of cardioversion within the first 4 weeks were calculated for an average person of both men and women, using results from the baseline table and the odds ratio. Risk of a composite event and risk of readmission with atrial fibrillation were presented as cumulative incidences curves with 95% CI, and as time dependent Cox proportional-hazard analyses adjusted for age, sex, comorbidities and concomitant drugs. The mean follow up time was 28.9 weeks in the dabigatran group and 33.6 weeks in the warfarin group; hence, a 30 weeks follow-up period was chosen. Sensitivity analyses were completed with age and sex matched groups excluding patients with ischemic heart disease and chronic heart failure and analysis for treatment bias to warfarin and dabigatran was performed. P-value of <0.05 was considered significant. For data management and analysis we used R version 3.1.1 (The R Foundation for Statistical Computing).

### Ethics

In Denmark, retrospective register studies do not require approval from the ethics committees. The Danish Data Protection Agency approved this study (Ref.no: 2007-58-0015 / GEH-2014-013 and I-Suite no: 02731) and data were made available to us in an anonymized format such that individuals could not be identified.

## Results

**Fig 1:**
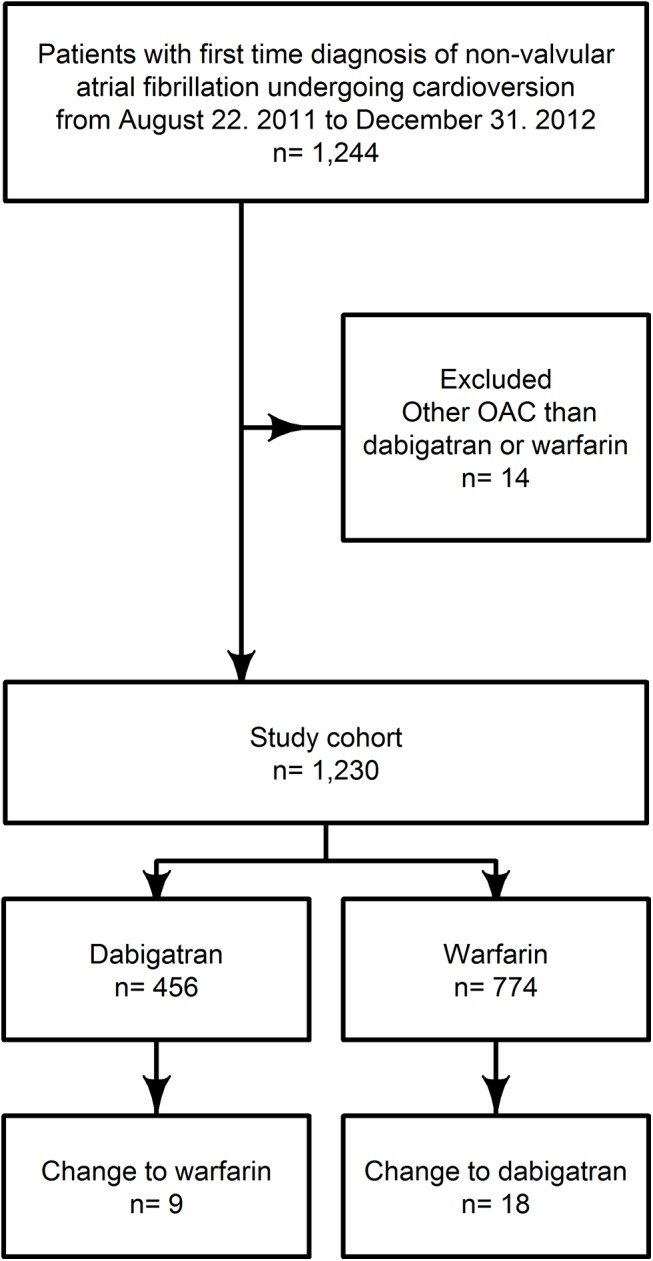
Flowchart of the patient population.

During the study period 1,230 patients were eligible to enter the study cohort, with 37% in the dabigatran group (n = 456), and 63% in the warfarin group (n = 774). Only 27 patients (2.3%) changed OAC regime in the follow up period. Selection of the study cohort is depicted in Fig 1. Chronic heart failure, hypertension and ischemic heart disease, antiplatelet usage was predominant in the warfarin group (Table 1).

**Table 1:** Baseline characteristics of patients by anticoagulation treatment status.

Characteristic	Dabigatran	Warfarin	p–value †
Number	456	774	
Age, * y (IQR)	66.3 (59.3–72.6)	67.4 (60.8–72.5)	0.046
Men, %	332 (72.8)	569 (73.5)	0.838
Stroke, (%)	27 (5.9)	45 (5.8)	1.000
Chronic Heart Failure, (%)	50 (11.0)	148 (19.1)	<0.001
Diabetes Mellitus, (%)	42 (9.2)	96 (12.4)	0.105
Liver disease, (%)	4 (0.9)	10 (1.3)	0.589
Bleeding, (%)	42 (9.2)	67 (8.7)	0.821
Hypertension, (%)	291 (63.8)	546 (70.5)	0.017
Ischemic Heart Disease, (%)	38 (8.3)	107 (13.8)	0.005
Peripheral arterial disease, (%)	5 (1.1)	17 (2.2)	0.187
Any Cancer, (%)	45 (9.9)	93 (12.0)	0.290
Chronic Kidney Disease, (%)	10 (2.2)	24 (3.1)	0.375
Chronic Obstructive Pulmonary Disease, (%)	31 (6.8)	43 (5.6)	0.447
Percutaneous Coronary Intervention, (%)	20 (4.4)	49 (6.3)	0.192
Coronary Artery Bypass Grafting, (%)	8 (1.8)	22 (2.8)	0.316
CHA2DS2-VASc, n (%)			0.103
0	68 (14.9)	83 (10.7)	
1	125 (27.4)	181 (23.4)	
2	133 (29.2)	247 (31.9)	
3	88 (19.3)	176 (22.7)	
≥ 4	42 (9.2)	87 (11.2)	
Verapamil, (%)	15 (3.3)	25 (3.2)	1.000
Amiodarone, (%)	26 (5.7)	38 (4.9)	0.595
Flecainide, (%)	5 (1.1)	3 (0.4)	0.155
Acetylsalicylic acid, (%)	129 (28.3)	252 (32.6)	0.106
Digoxin, (%)	160 (35.1)	269 (34.8)	0.950
Antiplatelet, ‡ (%)	14 (4.7)	53 (10.2)	0.008
NSAID, § (%)	64 (14.0)	123 (15.9)	0.427

* Continuous variables are presented as median (interquartile range [IQR])

† The P value is for the comparison between groups and is based on the Fisher’s exact test or Chi-Square test for categorical variables and Kruskal-Wallis for continuous variables.

‡ Clopidogrel, presugrel or ticagrelor.

§ Diclofenac, ibuprofen, celecoxib, rofecoxib, naproxen.

### Time to cardioversion and cardioversion within the first 4 weeks

**Fig 2:**
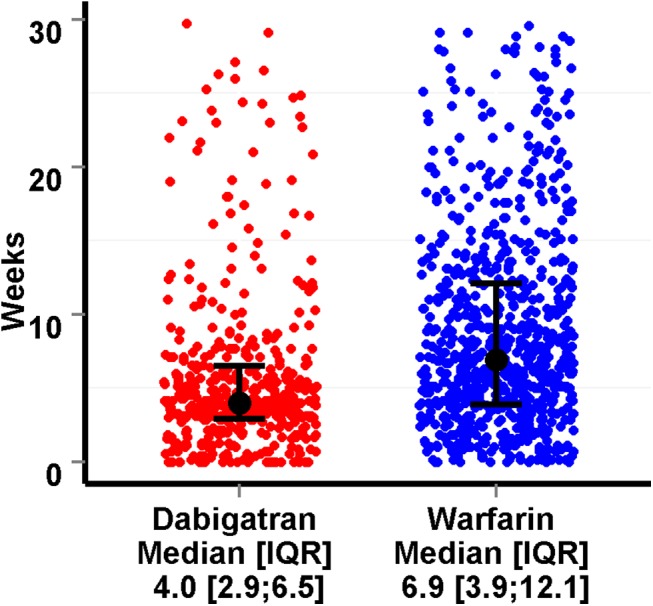
Jitter plot with weeks from claimed prescription of anticoagulation treatment, to cardioversion in the two groups, with median weeks and interquartile range (IQR). One dot represents one patient.

The median time to cardioversion were 4.0 (IQR 2.9 to 6.5) and 6.9 (IQR 3.9 to 12.1) weeks in the dabigatran and warfarin groups respectively (Fig 2). Significantly more patients in the dabigatran group (n = 229; 50%) underwent cardioversion within the first 4 weeks, compared to the warfarin group (n = 207; 27%), p<0.005. The adjusted odds ratio of cardioversion within the first 4 weeks was 2.3 (95% CI 1.7 to 3.1) in favor of dabigatran treatment, with transesophageal echocardiogram performed prior to the cardioversion in 26 (6%) and 40 (5%) in the dabigatran and warfarin group, respectively (Table 2). Predicted probability of cardioversion within the first 4 weeks in 66 years old men with hypertension were 43.8% and 25.4% in the dabigatran and warfarin group respectively, and 47.1% and 27.9% for a 66 years old woman with hypertension (Table 2).

**Table 2:** Cardioversion within the first four weeks.

	Dabigatran	Warfarin	P—Value
Incidence of Cardioversion within the first four weeks, (%)*	229/456 (50%)	207/774 (27%)	<0.005
Odds ratio and 95% CI of cardioversion within the first four weeks, †	2.3 (1.7–3.1)	1 (ref.)	<0.005
Transesophageal echocardiogram, (%) ‡	26 (6%)	40 (5%)	0.696
Predicted probability of cardioversion within the first four weeks			
66 years old men with hypertension, §	43.8%	25.4%	
66 years old women with hypertension, §	47.1%	27.9%	

* The P value is for the comparison between groups and is based on the Chi-Square test

† Odds ratio was adjusted for age, sex, diabetes, ischemic heart disease, chronic heart failure, liver disease, bleeding, cancer, chronic kidney disease, chronic obstructive pulmonary disease, stroke, peripheral arterial disease, dronedarone, amiodarone, flecainide, verapamil, antiplatelets, NSAID and acetylsalicylic acid.

‡ Fisher's exact test.

§ Predicted probabilities are calculated from for characteristics of an average person of both sexes as found in Table 1.

### Composite safety endpoint

**Fig 3:**
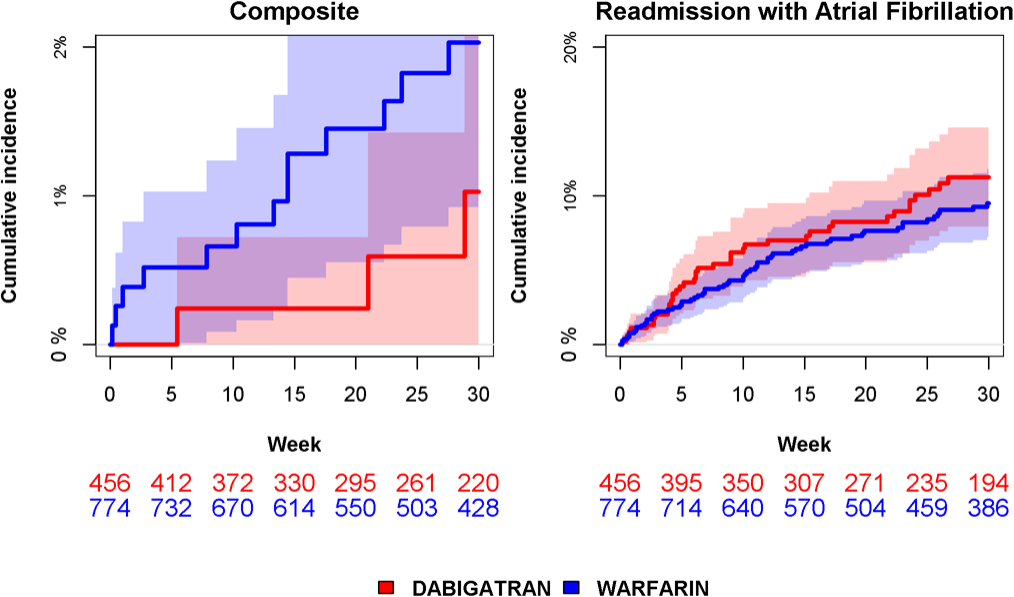
Cumulative incidence and patients at risk within 30 weeks from cardioversion with dabigatran and warfarin treatment. Left: Composite endpoint of stroke, bleeding and death. Right: Readmission with atrial fibrillation. Shades represent 95% confidence intervals. At risk table represents number of patients at risk of event at given time.

The composite endpoint of stroke, major bleeding or death within 30 weeks after cardioversion occurred in 3 (0.7%; Bleed 0, Stroke 1; Death 2) in the dabigatran group and in 13 (1.4%; Bleed 0, Stroke 1, Death 12) patients warfarin groups. Of the 465 patients in the dabigatran group, nine patients switched to warfarin treatment and were censored at the day switching to warfarin. In the warfarin group 18 changed to dabigatran and was censored at the day switching to dabigatran (Table 1). There was an even distribution of cumulative incidence over time with 2.0% and 1.0% after 30 weeks in the warfarin and dabigatran groups, respectively (Fig 3). The time dependent Cox regression analysis found a hazard ratio (HR) of 1.33 (95% CI 0.33 to 5.42) for the composite endpoint in the warfarin group compared to the dabigatran group.

### Readmission with atrial fibrillation

Readmission with atrial fibrillation within 30 weeks after cardioversion was experienced by 38 (8.3%) in the dabigatran group and by 59 (7.6%) patients in and warfarin group. The cumulative incidences of readmission with atrial fibrillation after 30 weeks were 9% and 11% in the warfarin and dabigatran groups, respectively (Fig 3). In the time dependent adjusted Cox regression analysis, there was no significant difference between the two groups regarding frequency of readmission with atrial fibrillation with a HR 0.66 (95% CI 0.41 to 1.08).

### Sensitivity analysis

In a sensitivity analysis we excluded patients with either ischemic heart disease or chronic heart failure, and propensity score matched the dabigatran group to the warfarin group with age and sex. The results were similar to the unmatched population, both regarding time to cardioversion (weeks (IQR) 3.9 (2.5 to 5.9) for dabigatran and 6.5 (3.6 to 11.9) for warfarin; p<0.005), chance of cardioversion within the first 4 weeks (OR (95%CI) 3.1 (2.3 to 4.2)), risk of composite events (HR (95%CI) 1.0(0.6 to 1.6)), and risk readmission with atrial fibrillation (HR (95%CI): 0.8 (0.1 to 4.5)).

## Discussion

In this nationwide study we investigated time to cardioversion according to oral anticoagulation treatment in patients with first-time atrial fibrillation. The main results of the study were that dabigatran allows shorter time to cardioversion than warfarin, and appears to be as effective and safe treatment strategy. This suggests that dabigatran might be favored over warfarin in patients where cardioversion is intended. This study is, to our knowledge, the first to investigate time to cardioversion in a real life population treated with dabigatran, whereas prior studies have been either randomised controlled trial or post hoc analysis with focus on rivaroxaban or apixaban.

The X-VeRT (eXplore the efficacy and safety of once-daily oral rivaroxaban for the prevention of caRdiovascular events in patients with non valvular aTrial fibrillation scheduled for cardioversion) study was a clinical trial, of patients with non-valvular atrial fibrillation randomized to rivaroxaban or warfarin prior to cardioversion. The duration of rivaroxaban and warfarin treatment prior to cardioversion was 3 and 4 weeks respectively.[12]

In a post hoc analysis of the ARISTOTLE (Apixaban for Reduction in Stroke and Other Thromboembolic Events in Atrial Fibrillation) trial, the time from study entry to cardioversion was analyzed in patients randomized to with either warfarin or apixaban treatment. The duration of apixaban and warfarin treatment prior to cardioversion was 36 and 35 weeks, respectively.[13]

In a post hoc analysis of the RE-LY (The Randomized Evaluation of Long-Term Anticoagulation Therapy) trial the risk for cardiovascular complications with either warfarin or dabigatran treatment were analyzed in patients who underwent cardioversion.[8] Time to cardioversion was not investigated in this study. The variation between the results found in our study, the X-VeRT study and the ARISTOTLE post hoc analysis can be explained by difference in both selection of study cohorts and in study designs.

In both the RE-LY and ARISTOTLE post hoc analyses, and in the X-VeRT study the rates of stroke and systemic embolism at 30 days was <1% and did not differ whether the patient was treated with a novel oral anticoagulant (NOAC) or a vitamin K antagonist. The rates of major bleeding ranged from 0.2% to 1.7% in the NOAC groups, and ranged from 0.3% to 0.6% in the Vitamin K antagonist groups.[8,12,13] All-cause mortality rates were 0.5% in the NOAC groups and 0.6% in the warfarin groups in the X-VeRT study and ARISTOTLE trial. And in the post hoc RE-LY trial seven died but this was not specified by group. In our study 0.7% in the dabigatran group and 1.4% in the warfarin group had a composite event of stroke, bleeding or all-cause mortality with no significant difference, making our findings comparable to the prior studies. The duration of atrial fibrillation episode increase the risk of recurrent atrial fibrillation in patients with intermitting atrial fibrillation.[14]

Despite three weeks longer duration of time to cardioversion in the warfarin group compared with the dabigatran group, the cumulative incidence of readmissions with atrial fibrillation was similar in the two groups. Similar results were found in the ACUTE (Assessment of Cardioversion Using Trans-esophageal Echocardiograph) study, where one group had only 3 days to cardioversion versus 31 days in the comparing group, but the same maintenance of sinus rhythm was found after 8 weeks in the two groups.[15] This suggests that the duration of atrial fibrillation time periods investigated in our and the ACUTE study are not long enough to increase the risk of recurrent episodes.

The post hoc analysis of both the ARISTOTLE and RE-LY study both investigated the safety of cardioversion in patients already in anticoagulation treatment; whereas the aim of the X-VeRT study was to investigate the safety of anticoagulation treatment in patients scheduled for elective cardioversion. Our results are therefore more comparable to the X-VeRT study than the two post hoc analyses, since approximately 50% of the patients in the dabigatran group in our study received cardioversion already within 4 weeks.

### Limitations

The main limitation is inherited in the observational design of the study and lack of clinical information. In addition information regarding the date of referral to cardioversion was unavailable, and intention of anticoagulation treatment regarding cardioversion was also unknown. Exact referral date to cardioversion would have made our study more comparable to randomized controlled trials. Furthermore, the treatment groups were not randomly assigned to either dabigatran or warfarin, increasing risk of selection bias to the groups, although this risk was limited according to our preliminary analysis as described previously.

## Conclusion

In this nationwide study, we found time to cardioversion shortened by 3 weeks with warfarin compared with dabigatran treatment, with more twice the chance of cardioversion within the first 4 weeks. We found no difference regarding risk of subsequent death, stroke, bleeding, or readmission with atrial fibrillation within 30 weeks after cardioversion.

## Appendix

**Appendix 1:** 

ICD-10 codes	
Atrial Fibrillation	I48
Rheumatic Heart Valve Disease	I05, I06, I080, I069, I081, I082, I083, DT823A,I068, I080A, I082A, I081A
Stroke	I63, I64, DG458, DG459
Bleeding	I60, I61, I690, I691, N02, R31, K228F, K251,K252, K254, K256, K260, K262, K264, K266,K270, K272, K274, K625, K633, K638B,K638C, K850, K922
Chronic Heart Failure	I50, J819
Ischemic Heart Disease	I21, I22
Atherosclerosis	I74
Cancer	C
Chronic Kidney Disease	I12, E102, E112, E132, E142, N03, N04, N05,N06, N07, N08, N11, N13, N14, N18, N19, N25,N26, N27, N28, N29, N391, Q61.
Chronic Obstructive Pulmonary Disease	J42, J43, J44
Liver disease	K7
ATC	
Warfarin	B01AA03
Marcoumar	B01AA04
Dabigatran	B01AE07
Rivaoxaban	B01AF01
Diabetes Mellitus	A10
Hypertension as usage of two different drugs classes.	C09, C03AA, C03C, C07A C02AB
Verapamil	C08DA
Amiodarone	C01BD
Fleccanide	C01BC
Acetylsalicylic acid	B01AC
NSAID	M01AB05, M01AE01, M01AH01, M01AH02, M01AE02
Antiplatelet	B01AC04, B01AC24, B10AC22
Digoxine	C01AA
Procedure Codes	
Cardioversion	BFFA0, BFFA00, BFFA01, BFFA04
Transesophageal echocardiogram	UXUC81, UXUC81C
Percutaneous Coronary Intervention	KFNG00, KFNG02, KFNG05, KFNG10, KFNG12, KFNG20, KFNG30, KFNG40, KFNG96
Coronary Artery Bypass Grafting	KFNA00, KFNA10, KFNA20, KFNA96, KFNB00, KFNB96, KFNC10, KFNC20, KFNC30, KFNC40, KFNC50, KFNC60, KFNC96, KFND10, KFND20, KFND96, KFNE00, KFNE10, KFNE20, KFNE96,
Prosthetic Valve Replacement	KFKD00, KFMA20, KFMA32A, KFMD00, KFMD33, KFMD96
